# Successful treatment of ceftazidime/avibactam combined with aztreonam in the NDM-producing Klebsiella pneumoniae bloodstream and intestinal infections in a NK/T lymphoma patient with agranulocytosis during autologous hematopoietic stem cell transplantation: a case report

**DOI:** 10.1007/s10096-022-04523-3

**Published:** 2022-11-12

**Authors:** Shiyi Liu, Qingqing Lin, Lizhi Ouyang, Chengjie Zhou, Huajun Wang

**Affiliations:** 1 Department of Critical Care Medicine, The Affiliated People’s Hospital of Ningbo University, Ningbo, Zhejiang China; 2 Department of Hematology, The Affiliated People’s Hospital of Ningbo University, Ningbo, Zhejiang China

**Keywords:** Carbapenem-resistant Klebsiella pneumoniae, Ceftazidime/avibactam aztreonam, New Delhi metallo-beta-lactamase

## Abstract

New Delhi metallo-beta-lactamase (NDM)-producing Klebsiella pneumoniae is increasingly reported worldwide. Clinicians face significant challenges in the treatment of this multidrug-resistant bacterium. The combination of ceftazidime/avibactam (CAZ/AVI) and aztreonam (ATM) is currently probably the most effective strategy for the treatment of such infection. We described a patient diagnosed with NK/T cell lymphoma who underwent autologous hematopoietic stem cell transplantation (ASCT) in the hematology department. The patient developed severe infection after ASCT. Blood and stool cultures showed carbapenem-resistant *K. pneumoniae*. Blood sample was detected as NDM-producing *K. pneumoniae*. We successfully treated this infection with CAZ/AVI and ATM.

## Introduction 

With the worldwide prevalence of multidrug-resistant bacteria, people have faced increasing concerns about infections that are not treated with susceptible antibacterial agents. According to data from the US Centers for Disease Control and Prevention, the infection rate of carbapenem-resistant Enterobacteriaceae (CRE) was 57/100,000, and the in-hospital mortality rate of nosocomial infection caused by CRE is 33.50%, among which the mortality rate of blood flow infection caused by CRE is as high as 43.10% [1]. CRE infection imposes a heavy burden on patients, and the cost of treatment is higher than the annual cost of many acute and chronic diseases [2]. Due to the use of antibiotics in the early stage and the inhibition of immune function after chemotherapy, the detection rate of carbapenem-resistant *K. pneumoniae* (CRKP) in patients with hematological malignancies is increasing, which has seriously endangered the life of the patients [3].

New Delhi metallo-beta-lactamase (NDM)-producing *K. pneumoniae* was first identified in India [4]. Ceftazidime/avibactam (CAZ/AVI) can be used to treat CRKP infection, but the drug lacks activity against NDM. Whereas aztreonam (ATM) is hydrolytically stable to class B MBL [5, 6]. The combination of ATM and CAZ/AVI has been reported to be effective for the treatment of infections caused by Enterobacteriaceae producing MBL. We describe the successful treatment of a patient with the CAZ/AVI and ATM combination below.

## Case presentation

In August 2021, a 62-year-old man diagnosed with NK/T-cell lymphoma was hospitalized in the hematology department for autologous hematopoietic stem cell transplantation (ASCT). On the 3^rd^ day after ASCT, the patient developed fever and diarrhea. We gave imipenem/cilastatin 500 mg Q6H IV and teicoplanin 400 mg Q12H IV for empirical anti-infection treatment. After 2 days of treatment, the patient still had a high fever, and watery yellowish stool more than 10 times per day. On the 5^th^ day after ASCT, the antibiotic regimen consisting of imipenem/cilastatin and teicoplanin was replaced by tigecycline 100 mg Q12H IV and cefoperazone/sulbactam 2.0 g Q8H IV. On the 6^th^ day after ASCT, the patient was transferred to ICU due to septic shock.

**Fig. 1:**
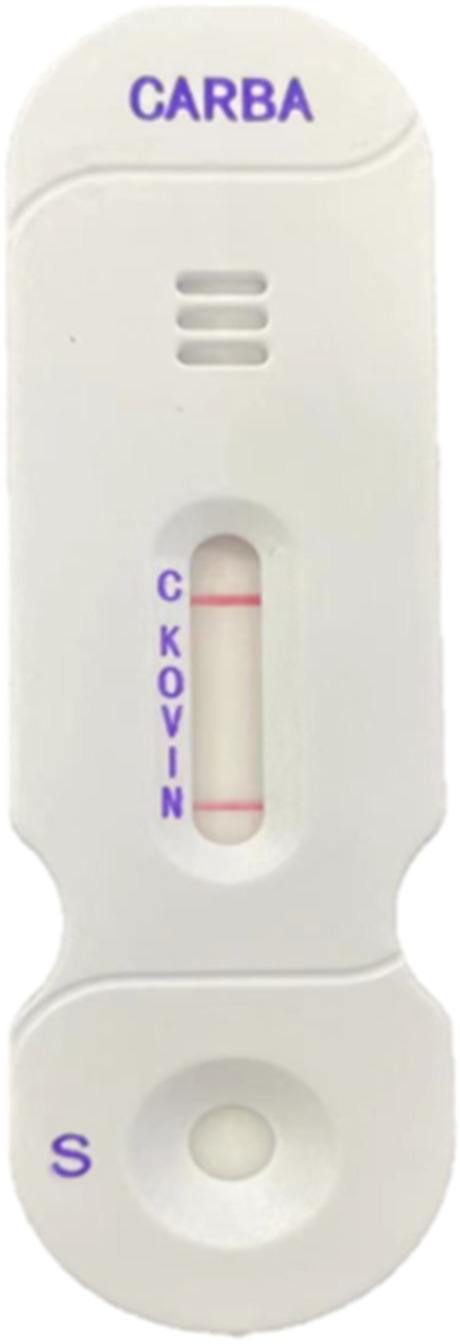
CARBA5 carbapenem detection reagent

We administered imipenem/cilastatin 500 mg Q4H IV and CAZ/AVI 2.5 g Q8H IV and teicoplanin 600 mg Q12H IV to anti-infective therapy. On the 3^rd^ day of hospitalization in the ICU, *K. pneumoniae* was detected in blood culture (Table 1) and stool culture (Table 2) obtained from the patient on 6^th^ after ASCT. Blood sample was detected to produce NDMCRKP by CARBA5 carbapenem detection reagent (Fig. 1). We started ATM 2.0 g Q8H IV and CAZ/AVI 2.5 g Q8H IV as antibiotic regimen. The patient’s clinical condition improved quickly after the introduction of this treatment regimen.

**Table 1:** Resistance profile of Klebsiella pneumoniae strains found in blood samples

Antibiotics	Result	MIC
Amikacin	Susceptible	≦2
Aztreonam	Resistant	32
Ceftazidime	Resistant	≧64
Cefoperazone/sulbactam	Resistant	≧64
Ciprofloxacine	Resistant	≧4
Doxycycline	Resistant	≧16
Cefepime	Resistant	≧32
Imipenem	Resistant	≧16
Levofloxacin	Resistant	≧8
Meropenem	Resistant	≧16
Minocycline	Resistant	≧16
Ceftazidime/Avibactam	Resistant	
Tobramycin	Resistant	≧16
Cotrimoxazole	Resistant	≧320
Piperacilline/Tazobactam	Resistant	≧128
Polymyxin E	Susceptible	≦0.5

**Table 2:** Resistance profile of Klebsiella pneumoniae strains found in stool samples

Antibiotics	Result	MIC
Amikacin	Susceptible	8
Aztreonam	Resistant	32
Ceftazidime	Resistant	≧64
Cefoperazone/sulbactam	Resistant	≧64
Ciprofloxacine	Resistant	≧4
Doxycycline	Resistant	≧16
Cefepime	Resistant	≧32
Imipenem	Resistant	≧16
Levofloxacin	Resistant	≧8
Meropenem	Resistant	≧16
Minocycline	Resistant	≧16
Ceftazidime/Avibactam	Resistant	
Tobramycin	Resistant	≧16
Cotrimoxazole	Resistant	≧320
Piperacilline/Tazobactam	Resistant	≧128
Polymyxin E	Susceptible	≦0.5

## Discussion

New Delhi metallo-beta-lactamase-1 (NDM-1) is a metal-ion-dependent β-lactamase (MBL), and bacteria carrying this enzyme can hydrolyze almost all β-lactam antibiotics, but ATM cannot form a productive complex with MBLs. ATM is a monocyclic β-lactam antibiotic that is active against MBL-producing enzymes, but can be hydrolyzed by extended-spectrum β-lactamases. Avibactam can protect ATM from being hydrolyzed by class A and class C β-lactamases, thereby retaining the attack activity of ATM against class B metalloenzyme-producing Enterobacteriaceae [7].

Marshall et al. demonstrated coordinated bactericidal effects of CAZ/AVI in combination with ATM against multiple Enterobacteriaceae with MBLs in 17/21 isolates. In a murine neutropenic thigh infection model, compared with CAZ/AVI alone, CAZ/AVI plus ATM reduced CFUs by almost 4log_10_ at 24 h post-dose [7]. Maraki and his colleagues assessed the in vitro synergy of ATM in combination with CAZ/AVI against 40 MDR, MBL-producing, and serine-β-lactamase-producing *K. pneumoniae* strains by an Etest-based synergy approach, with synergy observed in 97.5% of the CAZ/AVI-ATM combination group [8]. Jayol A et al. recovered 63 K*. pneumoniae* isolates from clinical samples. Among them, 15 strains are NDM-like producers. The combination of CAZ/AVI and ATM was found to be particularly effective against NDM-producing *K. pneumoniae* using MIC test strips [9]. Falcone et al. found that CAZ/AVI combined with ATM (19.2%) had a lower 30-day mortality rate than other single antibiotics or combinations (44%) in a prospective study [10].

Bocanegra-Ibarias et al. reported a case of aplastic anemia patient with agranulocytosis and bloodstream infection after chemotherapy. The blood culture result was *K. pneumoniae* with NDM-1, which was successfully treated with ATM and CAZ/AVI [11]. Perrotta and colleagues also reported successful treatment of *K. pneumoniae* NDM sepsis and intestinal decolonization with ATM in combination with CAZ/AVI in a patient with TTP and SARS-CoV-2 nosocomial infection [12].

Currently, there are few case reports on CAZ/AVI combined with ATM in the treatment of MBL-producing *K. pneumoniae* infection in China. The CAZ/AVI + ATM combination overcame MBL/NDM producing *K. pneumoniae* by bypassing the most important resistance determinants.

## Conclusions

CAZ/AVI combined with ATM is currently an effective treatment option for MBL-producing microbial infections.

## Data Availability

Not applicable.
